# Causes of Mortality and Diseases in Farmed Deer in Switzerland

**DOI:** 10.4061/2010/684924

**Published:** 2010-07-15

**Authors:** Veronika Sieber, Nadia Robert, Martina Schybli, Heinz Sager, Raymond Miserez, Monika Engels, Marie-Pierre Ryser-Degiorgis

**Affiliations:** ^1^Centre for Fish and Wildlife Health, Institute of Animal Pathology, Vetsuisse Faculty, University of Bern, Postfach 8466, 3001 Bern, Switzerland; ^2^Institute of Parasitology, Vetsuisse Faculty, University of Bern, Länggassstrasse 122, 3012 Bern, Switzerland; ^3^Institute of Veterinary Bacteriology, Vetsuisse Faculty, University of Bern, Postfach 8466, 3001 Bern, Switzerland; ^4^Institute of Veterinary Virology, Vetsuisse Faculty, University of Zurich, Winterthurerstrasse 266a, 8057 Zurich, Switzerland

## Abstract

To investigate diseases and causes of mortality in Swiss farmed deer, deer found dead or shot due to diseased condition between March 2003 and December 2004 were requested for a complete postmortem examination. One hundred and sixty-two animals were submitted. Perinatal mortality, necrobacillosis in 3 week to 6 month old deer, and endoparasitosis in 6 month to 2 year old deer were identified as the most important causes of loss, followed by ruminal acidosis, which was diagnosed in 22% of deer older than 1 year. Congenital malformations were observed in 15% of deer less than 6 months old. Reportable infectious diseases known as major problems in deer farming in other countries were rare (yersiniosis, malignant catarrhal fever) or not observed (tuberculosis, chronic wasting disease). Overall, the results indicate that the Swiss deer population does not present major health problems of concern for domestic animals.

## 1. Introduction

Deer farming is a growing business worldwide. In Switzerland, the farmed deer population has been constantly increasing for the past 25 years, ever since the Swiss Federal Office for Agriculture officially started promoting deer farming [1]. According to a questionnaire survey, diseases are a common cause of loss on deer farms [2]. However, except for endoparasites, which have been reported as a common health problem [3], and a study indicating the absence of tuberculosis [2], no data are available concerning the spectrum of diseases in farmed deer in Switzerland.

Disease surveillance is compulsory neither in free-ranging nor in captive wild species in Switzerland, and data on the occurrence of reportable diseases in farmed deer are lacking. However, it is known from other countries that infections such as yersiniosis, listeriosis, tuberculosis, malignant catarrhal fever, paratuberculosis, and chronic wasting disease can cause high losses in farmed deer [4–10]. Deer herds often live in vicinity of domestic farm animals. Thus, interspecific transmission of pathogens has to be considered. Because the Swiss domestic farm animal population is free of major reportable diseases, it is of concern whether Swiss farmed deer could play a role as reservoir for infectious diseases affecting domestic livestock. 

The aim of this study was to investigate causes of morbidity and mortality in Swiss farmed deer, with special attention to reportable diseases.

## 2. Materials and Methods

### 2.1. Study Population and Material Collection

The whole farmed deer population of Switzerland was considered for this study. Nowadays, about 9400 deer, thereof 5200 breeding animals, are kept in 432 farms (Status January 2005) [11]. Eighty-five percent of the farms keep fallow deer (*Dama dama*), 8% red deer (*Cervus elaphus elaphus*), 3% sika deer (*Cervus Nippon*), and 1% wapiti (*Cervus elaphus nelsoni*). Most herds count about 20 to 40 breeding females, their fawns, and one or two breeding males. According to Swiss legislation, deer farmers have to be officially registered. All farmers were asked to send all deer found dead or shot because of disease symptoms for costless pathological investigation from March 2003 to December 2004. Participation was on a voluntary basis, and for laboratory analysis and data management, anonymity of the participants was preserved.

### 2.2. Pathological Examination

A full necropsy was performed immediately after reception. Tissue samples of all inner organs were systematically collected for histology. Samples were fixed in 4% buffered formalin, embedded in paraffin, cut at 4 *μ*m and mounted on positively charged glass slides (Super-Frost, Menzel-Gläser, Germany), then stained with haematoxylin-eosin (HE), and analysed by light microscopy. For fawns and juveniles presenting diarrhoea or wasting, and all older animals, a Ziel-Neelsen staining was performed on intestinal tissue to detect *Mycobacterium paratuberculosis*. If necessary to obtain a diagnosis, additional special stainings such as iron-stain, Van Giesson-stain, or PAS-stain were also applied.

### 2.3. Microbiology

Except for cases with clear noninfectious cause of death such as trauma, spleen, liver, kidney, and lungs were systematically submitted for bacteriological examination to the Institute of Veterinary Bacteriology in Bern. If pathological changes were present or suspected in the intestine, samples from the small and large intestines were also sent for bacteriological examination. Bacteriological analysis was done following routine diagnostic methods [12]. In one case, for which there was a suspicion of malignant catarrhal fever, a Polymerase Chain Reaction (PCR) for ovine herpes virus 2 was performed, using paraffin-embedded material [13, 14].

### 2.4. Parasitology

Faeces of deer >4 weeks old (*n* = 84) were systematically analysed for protozoan and helmintic parasites with a combined sedimentation-flotation technique using ZnCl_2_ and for lungworm larvae with the Baermann-Wetzel method [15]. Parasitic infestations were classified as mild (≤1 egg per microscopic field at a tenfold magnification), marked (2–9 eggs), or severe (≥10 eggs). For the detection of Cryptosporida, a modified Ziehl-Neelson stain was used [15].

### 2.5. Classification Criteria

Deer were classified in six age categories: (1) abortion (not at term), (2) neonates (from birth to 7 days old, including mature stillbirths), (3) fawns (>1 week to 6 months old), (4) juveniles (>6 months to <1 year old), (5) subadults (from 1 to <2 years old), and (6) adults (≥2 years old). If the owner did not know the date of birth, the age of animals in their first year of life was estimated considering the month of the year and normal birth period. Distinction between subadults and adults was made considering dentition characteristics [16].

Postmortem results were classified considering the etiology, as proposed by Euzenat [17]: The “cause of death” was defined as the event leading directly to the death of the animal, or the reason for euthanasia or shooting if the animal did not die spontaneously. If appropriate, a predisposing factor for the affection seen as cause of death was mentioned. Secondary findings were defined as lesions independent from the process of death and not severe enough to cause death. The cause of death was considered “indeterminable” if the pathological examination was not possible due to lack of organs or advanced autolysis, and “unclear” if it could not be identified despite thorough examination.

### 2.6. Statistical Analysis

Data management and descriptive statistics were conducted in Microsoft Excel 2000 (Microsoft Corporation, Redmond, Washington, USA). All statistical calculations were performed with NCSS 2001 Statistical Software (J. L. Hintze, Kaysville, Utah, USA). Statistical significance of differences was analysed using Fisher's Exact Test.

## 3. Results

### 3.1. Submitted Material

During the study period, 78 breeders (18.1% of all Swiss deer breeders) submitted at least one animal for investigation. Animals were submitted from 17 out of the 24 Swiss cantons (71%) having deer farms. Overall, 162 deer (141 whole carcasses, and selected organs from 21 animals) were analysed. One hundred and twenty-eight deer were found dead, 21 were shot due to disease or trauma, five were euthanized, and eight animals were stillborn or aborted. With regards to species, 86% (*n* = 140) were fallow deer, 7% (*n* = 11) red deer, 5% (*n* = 8) sika deer, 1% (*n* = 2) wapitis, and 0.6% axis deer (*n* = 1). Animals in the first year of life (neonates, fawns, and juveniles) represented 66% of the investigated material (Table 1). The overall sex ratio was 76 males (46.9%) to 86 females. In animals less than two years old, sex ratio was almost balanced with 45.5% females (*n* = 55), but in adults, there were more females (75.6%, *n* = 31) than males. This difference was statistically significant (*P* = .001).

### 3.2. Infectious Diseases

Causes of death of investigated deer are listed in Table 1. Infectious diseases (64.2%) outnumbered the noninfectious causes of death (25.9%). No significant difference was noted between the causes of mortality in males and females. In contrast, differences were obvious between age classes. Deer less than two years old (abortions excluded) were significantly more affected by infectious diseases than adults (*P* < .001). Bacterial diseases were more common in neonates and fawns than those in older age classes (*P* < .001 in all cases). Parasitic diseases affected juveniles more than the other age classes (*P* < .001 compared to fawns and adults, and *P* < .05 compared to subadults) but were also the predominant cause of death in subadults (50%).

Infectious diseases were mainly due to bacteria (68.3% or 71 out of 104 infectious cases) and lung and/or gastrointestinal parasites (29.8%). Necrobacillosis was the most common bacterial infection, affecting 17.3% of the investigated deer. Only fallow deer were affected, and only the oral form of the disease was observed. Fawns were significantly more affected by necrobacillosis (*n* = 24, 85.7%) than juveniles (*n* = 2; *P* < .001), subadults (*n* = 0; *P* < .001), and adults (*n* = 2; *P* < .001). The second most important bacterial infection was septicaemia caused by *Escherichia coli. *One case of yersiniosis (*Yersinia pseudotuberculosis*) was diagnosed in a fallow deer fawn.

Endoparasites due to gastrointestinal and/or lungworms (mostly *Dictyocaulus* sp.) was considered as cause of death in 31, as predisposing factor in two, and as a secondary finding in 39 deer (Table 3). *Trichuris* sp., *Capillaria* sp., and Trichostrongylidae were by far the most prevalent gastro-intestinal parasites, particularily in fatal cases. Liver flukes (*Dicrocoelium dendriticum*) were additionally identified in three animals from a single farm. Overall, 72 out of 84 deer tested by coprological methods (85.7%) were affected by endoparasites, including all juveniles and all subadults. The difference in prevalence was significantly higher in juveniles than in fawns (*P* < .001) and adults (*P* < .05).

A mycotic pneumonia (*Aspergillus* sp.) and malignant catarrhal fever (Ov. herpes virus 2) were diagnosed in an adult and a juvenile male sika deer, respectively. *Mycobacterium paratuberculosis* was not detected in any of the investigated deer.

### 3.3. Noninfectious Diseases

Ruminal acidosis was the second most common noninfectious cause of death after trauma. Overall, ruminal acidosis was observed in 11 deer, all over one year old, including 24% of the investigated adults. It was considered as cause of death in eight deer (16% of all animals over one year old, and 17% of adults only) and as secondary finding in three. Diagnosis of ruminal acidosis was based on a ruminal pH lower than six (*n* = 6) and/or histological lesions of the ruminal mucosa (*n* = 6) such as parakeratosis, small pustules, and hyperkeratosis. Except for a subadult male, all affected deer were adults. Adult males were more commonly affected (4/10, 40%) than adult females (6/31, 19%) but this difference was not significant (*P* = .22).

Congenital malformations were observed in 12 fallow deer less than one year old: two aborted foetuses, seven neonates, and three fawns. They were either considered as cause of death, as predisposing factor, or as a secondary finding (Table 2).

### 3.4. Secondary Findings

Overall, 24 different secondary findings (Table 3) were observed in 65/162 deer (40%). In addition, ectoparasites, such as ticks, fleas, and rarely mallophages, were observed in animals with a poor general condition.

*Clostridium perfringens* was isolated from 23 deer. Of these, nine showed pathological changes that could be related to the presence of C. *perfringens* (six deer out of eight with fatal ruminal acidosis, one with ruminal acidosis as a secondary finding, another with enteritis, and the last with colitis), and 14 (including neonates) showed no associated pathological lesions.

### 3.5. Neonatal Mortality

Perinatal mortality was identified as an important cause of loss and concerned mainly neonates. All investigated neonates were fallow deer. They were born during the normal birth period for the species, that is, from May until end of August, with a peak in June [18], but had low birth weights considering references given in the literature [19, 20] and observations of Swiss breeders [11]: mean weight was 3.4 kg (range: 1.9–5.5 kg), including 17 animals (45%) less than 3.4 kg. Losses were mainly due to bacterial infections (50%), usually septicaemia, and hypoxic shock (6/38; 16%; Table 1). A diagnosis of hypoxic shock was made if the animal presented both atelectatic lungs and aspiration in absence of other pathological findings.

Aspiration of amniotic fluid and/or meconium, alone (*n* = 9) or together with inanition (empty stomach) (*n* = 15) were often considered as a predisposing factor for disease in neonates (24/38, 63%). Aspiration (*n* = 6) and inanition (*n* = 1) were also diagnosed as secondary findings (7/38, 18%). Overall, 45% (17/38) of the investigated neonates had an empty stomach, and 79% (30/38) presented an aspiration. Aspiration was often associated with poorly unfolded lungs and atelectasis (*n* = 24, 63%; *P* < .001). Six breeders sent two to three neonates during the two years of investigation; all other neonates were single cases from different farms.

## 4. Discussion

The present study is the first to describe the health status of Swiss farmed deer in general. Three major causes of loss were detected in deer in their first year of life: perinatal mortality, necrobacillosis in fawns (59% of all fawns affected), and endoparasitosis in juveniles (86%). In older animals, health problems were more diverse. However, lethal endoparasites was frequent in subadults (50%), and ruminal acidosis was a common cause of death in adults (17%). These results are similar to data from Germany [10, 21] but clearly differ from reports from other continents. In Canada, necrobacillosis is an important cause of loss, but endoparasites and perinatal mortality are not mentioned as predominant problems [22]; furthermore, Chronic Wasting Disease is an important issue in this country [9]. In New Zealand and Australia, perinatal mortality is mentioned as a cause of loss [20] but reportable infectious diseases are the most important health problem, in particular tuberculosis, yersiniosis, and malignant catarrhal fever (MCF) [7, 23]. In the present study, only one case each of MCF and yersiniosis were identified in two years. This indicates that both diseases affect Swiss farmed deer but prevalences appear to be very low. These diseases are uncommon in Swiss domestic livestock, possibly explaining the rare occurrence in farmed deer. Furthermore, Chronic Wasting Disease does not seem to occur in Europe (Belgium [24], Germany [25], Switzerland [26]). We suggest that perinatal mortality, necrobacillosis, and endoparasites are not just problems typical of European countries, but that in North America, Australia, and New Zealand, they are overshadowed by the high prevalence of other contagious infectious diseases.

Composition of the study material was dependent on the breeder's compliance. Because participation was on voluntary basis, deer breeders did probably not send all dead animals for examination. Nevertheless, nearly a fifth of the Swiss breeders participated in the present study, and animals originated from most cantons having deer farms. The proportion of the different deer species and the sex ratios in the age groups in our study reflects well the actual Swiss farmed deer population. However, according to the very low proportion of adult males in the population (1 male for 20–40 females), males were rather overrepresented in our study (1 male for 3 females).

The prevalence of the different causes of death is certainly biased. First, causes of death easily diagnosable by breeders, such as trauma or predation, are surely underrepresented because these cases are usually not sent for examination. Results obtained in a questionnaire survey confirm this assumption [2]: breeders recorded trauma as cause of death in 14% and predation in 13% of dead deer, but only 20% of all dead animals were sent for pathological investigation. However, one can assume that farmers with important losses would have taken the opportunity to submit carcasses as the analyses were performed at no cost for the breeder. Thus, although the results of this study probably do not reflect the real proportions of all causes of mortality, infectious diseases with high prevalence within a herd or with sporadic but frequent occurrence within the entire Swiss deer population should have been identified.

A large part (23%) of the material investigated in this study consisted of neonates, indicating that mortality of deer in the first week of life represents an important source of losses in deer farming. All neonates but one were under the normal birth weight which is considered to be about 5 kg for fallow deer [19], and almost 50% weighed less than 3.4 kg, even though all were born during the normal fawning season. Those animals with very low birth weight were never observed standing, and/or pathological examination revealed findings such as an empty stomach, poorly unfolded lungs and/or aspiration of amniotic fluid and meconium. Aspiration of foreign material during birth and/or poorly unfolded lungs can cause death directly (hypoxic shock) or indirectly by weakening the animal and leading to lack of colostrum ingestion (inanition) and high susceptibility to bacterial infections. Indeed, most neonates of this study died of hypoxic shock and/or bacterial infection. Aspiration of amniotic fluid and meconium is generally suggestive of dystocia [27]. However, dystocia is only described in deer giving birth to offspring of too large size compared to the mother [20]. Thus, we postulate that aspiration of amniotic fluid in underweight newborn deer might be due to prolonged birth process in relation with an insufficient condition of the dam.

Several studies on farmed and free-ranging deer of different species also report an important perinatal mortality [20, 28–30]. Significant differences between years are common [29, 30]. Age, weight, and condition of the does, quantity and quality of feed, weather (temperature, humidity, wind, exposure to sun), and management (appropriate shelter and fawning area) are seen as important factors influencing perinatal mortality [31]. Essentially, nonviable fawns born at the right time of the year appear to be due to underweight or yearling mothers [31–33]. Furthermore, a low birth weight has been identified as a major cause of loss in newborn fallow and red deer [20, 29, 30, 32]. English and Mulley [20] propose a minimal birth weight of 3.4 kg as the minimum for a fallow deer fawn to be able to stand and suckle and therefore to survive the first few days. In addition, these authors suggest that does abandon their weak fawns, thus considering mismothering of fawns with a low birth weight as a direct cause of perinatal mortality. In the present study, most cases of perinatal mortality were single events in a herd, and we therefore suggest that the observed perinatal mortality lies within normal range. However, in farms with high perinatal mortality, body condition of does and further factors such as weather conditions during birth period should be examined.

Necrobacillosis is a common bacterial disease in young deer fawns [5, 21, 34, 35]. It is believed that at this age, deer are particularly prone to small mouth injuries due to teething and weaning and are therefore more exposed to an infection. Necrobacillosis is furthermore considered a multifactorial disease associated with a number of risk factors including food composition and holding conditions [5, 21, 34], and appropriate management strategies seem the only way to prevent and control the disease in deer farming.

Endoparasites is obviously an important problem in deer in their first two years of life, especially between 6 and 12 months old [3, 8, 10, 21]. Weak, malnourished individuals are at higher risk for severe endoparasites, and adequate supplementary feeding is believed to prevent losses due to starvation and parasites during winter [5, 36–38]. Besides a possible insufficient immunity in early life, juvenile deer often do not get enough antiparasitic drugs for an efficient treatment due to the hierarchy in the herd. Thus, implementation of adequate feeding strategies and separate deworming of fawns and juveniles should noticeably reduce losses due to endoparasites on deer farms [3, 36–38].

Ruminal acidosis was the second most common noninfectious cause of death after trauma, and it was also observed as a secondary finding. Overall, almost 25% of all adult deer were affected. This metabolic disease has been reported in farmed deer in America [39], in Germany [10, 21], and in the United Kingdom [5]. Matzke [21] and Müller et al. [10] describe both abrupt changes of diet or inadequate feeding as a cause, as it is known in cattle [40]. In some Swiss deer farms, bread (donated by visitors or the farmer himself) is still fed in too large amounts for a ruminant. Furthermore, dominant animals are particularly at risk when eating too large portion of pellets or supplementary food. This could explain the higher prevalence of affected males compared to females in our material.

In the present study, *C. perfringens* was found in the abomasum and intestine of seven animals with ruminal acidosis, two with enteritis or colitis, and 14 neonates without associated lesions. Clostridia can lead to disease by causing enteritis, diarrhoea with only minor mucosal lesions, or enterotoxaemia, but bacteria alone are not pathogenic and are commonly found in the gut in a variety of circumstances, where they cannot be implicated as aetiologic agent; exotoxins are required to induce disease [41]. The pathogenesis of enteric infection with *C. perfringens* requires a change in the enteric microenvironment favorable to massive expansion of luminal populations of clostridia, such as a change in feed and abnormally nutrient-rich ingesta, and invasion of the host via the damaged ruminal mucosa can lead to a fulminant toxic shock [40, 41]. Thus, the association of ruminal acidosis and *C. perfringens* is not surprising, as ruminal acidosis might lead to death by predisposing affected deer to endotoxaemia. 

Congenital malformations were diagnosed several times either as cause of death, predisposing factor, or secondary finding. Four aborted foetuses showed skeletal deformities. Baker et al. [42] observed similar malformations of unknown aetiology in four farmed fallow deer neonates and described them as a form of chondrodystrophy. In the present study, histology could not be performed due to advanced decomposition, and lesions could not be further characterized. The congenital liver anomaly found in three neonates and one fawn seems to be similar to a disease observed in Australia [43]. The cause for this anomaly is not known, but Harper et al. [43] suspect a plant-intoxication during early pregnancy. The other malformations observed are known to occasionally occur in domestic ruminants and the low prevalences observed in this study are probably within normal range.

## 5. Conclusions

Reportable infectious diseases known as a problem in deer farming in other countries seem to be rare (yersiniosis, malignant catarrhal fever) or even absent (tuberculosis, chronic wasting disease) in Swiss farmed deer. The main health problems appear to be perinatal mortality, necrobacillosis, endoparasitosis, and to a lesser extent ruminal acidosis. These diseases can typically be influenced by management strategies, in particular by appropriate feeding. However, although these diseases appear at a high prevalence in our data set, they are not necessarily of economic importance for the deer breeders. Indeed, mostly single cases were observed within a herd, and only few breeders reported higher losses requiring management changes. Thus, results indicate that the Swiss farmed deer population does not present major health problems. Nevertheless, disease awareness of deer breeders is essential, and pathological investigation of unclear cases is further recommended.

## Figures and Tables

**Table 1:** Causes of death in farmed deer from Switzerland (2003-2004) in the different age classes.

Cause of death	Abortion	Neonate	Fawn	Juvenile	Subadult	Adult	Total	%
Trauma^(a)^		3		1		7	11	7
Metabolic dysfunction^(b)^					1	8	9	6
Circulatory collapse^(c)^/Vascular					1	5	6	4
Hypoxic shock		6					6	4
Malformation	2	2	1				5	3
Dehydratation^(d)^		1					1	1
Neoplasia^(e)^						2	2	1
Miscellaneous^(f)^		1				1	2	1

Total non-infectious	2	13	1	1	2	23	42	26

Bacterial		19	38	2	2	10	71	44
Necrobacillosis^(g)^			**24**	**2**		**2**	**28**	
Yersiniosis			**1**				**1**	
* E. coli* septicaemia		**8**	**1**				**10**	
Other septicaemia^(h)^		**8**	**9**		**2**	**3**	**21**	
Miscellaneous^(i)^		**3**	**3**			**5**	**11**	
Parasitic				24	5	2	31	19
Mycotic						1	1	1
Viral				1			1	1

Total infectious	0	19	38	27	7	13	104	64

Unclear	1	6	2	0	1	0	10	6
Indeterminable	1	0	0	0	0	5	6	4

Total	4	38	41	28	10	41	162	100

^(a)^Mainly due to injuries from stags, misadventure, or handling.

^(b)^Ruminal acidosis (8) and massive haemosiderosis due to an undefined problem in the iron household. (1)

^(c)^Causes: stroke of lightning (3); atherosclerosis, endocard, and myocard fibrosis (2); exhaustion (1).

^(d)^Cause: diarrhoea of unknown aetiology.

^(e)^One case each of bile duct carcinoma and salivary gland adenocarcinoma.

^(f)^One case each of inanition and chronic indigestion due to a foreign body in the rumen.

^(g)^Necrobacillosis was diagnosed in cases of anaerobic mixed flora cultured in correspondence with typical pathological findings.

^(h)^ *S* *treptococcus* sp. (6), *Pasteurella* sp. (2), *Aeromonas* sp. (2), *Enterobacter* sp. (2), and mixed flora (4), histological diagnosis without successful bacterial isolation (6).

^(i)^Multifocal purulent inflammation (1), purulent pneumonia (2), enteritis (2), colitis (3), metritis (2), alveolar periostitis (1).

**Table 2:** Congenital malformations in farmed deer from Switzerland (2003-2004) and their role in the process of death.

Malformation	Cause of death	Predisposing factor	Secondary finding
Skeletal malformation^(a)^	2	1	1
Palatoschisis		1	
Diaphragmal hernia	1		1
Liver malformation^(b)^	1		3
Ventricle septum defect	1		

^(a)^Shortened limbs and malformed cranium (2); contracted carpal joints alone (1) or together with malformed scapulae and humeri, and weakened bones with prenatal fractures (1).

^(b)^Biliary atresia with marked cholestasis and diffuse dissecting fibrosis.

**Table 3:** Secondary findings in farmed deer from Switzerland (2003-2004) in the different age classes.

Diagnosis	Abortion	Neonate	Fawn	Juvenile	Subadult	Adult	Total
Endoparasites			14	4	3	18	39
Mild aspiration of amniotic fluid		6	2				8
Beginning septicaemia	2	1		5			8
Aspiration pneumonia				1		4	5
Abomasal ulcer			3	1	1		5
Congenital malformation		3	2				5
Ruminal acidosis						3	3
Myocardial degeneration and tubulonephrosis			2				2
Parasitic dermatitis					1	1	2
Alveolar periostitis				1			1
Candidiasis (tongue)				1			1
Colitis						1	1
Purulent hepatitis				1			1
Endometrial hyperplasia						1	1
Inanition		1					1
Haemosiderosis			1				1
Omphalophlebitis			1				1
Bacterial pneumonia						1	1
Pyelonephritis		1					1
Unevenly worn cheek teeth						1	1
Teleangiectasia (liver)						1	1
Terminal thrombosis				1			1
Thymus involution		1					1
Chronic tonsillitis						1	1
